# Serum BDNF Concentrations Show Strong Seasonal Variation and Correlations with the Amount of Ambient Sunlight

**DOI:** 10.1371/journal.pone.0048046

**Published:** 2012-11-02

**Authors:** Marc L. Molendijk, Judith P. M. Haffmans, Boudewijn A. A. Bus, Philip Spinhoven, Brenda W. J. H. Penninx, Jos Prickaerts, Richard C. Oude Voshaar, Bernet M. Elzinga

**Affiliations:** 1 Institute of Psychology, Leiden University, Leiden, The Netherlands; 2 Leiden Institute for Brain and Cognition, Leiden University Medical Center, Leiden, The Netherlands; 3 Parnassia-BAVO Group, Department of Affective Disorders, Medical Chronobiology, PsyQ, The Hague, The Netherlands; 4 Department of Psychiatry Radboud University Medical Center, Nijmegen, The Netherlands; 5 Department of Psychiatry, Leiden University Medical Center, Leiden, The Netherlands; 6 Department of Psychiatry, EMGO Institute and Neuroscience Campus Amsterdam, VU, Amsterdam, The Netherlands; 7 University Center for Psychiatry, University Medical Center Groningen, University of Groningen, Groningen, The Netherlands; 8 Department of Psychiatry and Neuropsychology, Maastricht University, Maastricht, The Netherlands; Chiba University Center for Forensic Mental Health, Japan

## Abstract

Earlier findings show seasonality in processes and behaviors such as brain plasticity and depression that in part are regulated by Brain-Derived Neurotrophic Factor (BDNF). Based on this we investigated seasonal variation in serum BDNF concentrations in 2,851 persons who took part in the Netherlands Study of Depression and Anxiety (NESDA). Analyses by month of sampling (monthly *n*’s >196) showed pronounced seasonal variation in serum BDNF concentrations (*P*<.0001) with increasing concentrations in the spring-summer period (standardized regression weight (ß) = 0.19, *P*<.0001) and decreasing concentrations in the autumn-winter period (ß = −0.17, *P*<.0001). Effect sizes [Cohen’s *d*] ranged from 0.27 to 0.66 for monthly significant differences. We found similar seasonal variation for both sexes and for persons with a DSM-IV depression diagnosis and healthy control subjects. In explorative analyses we found that the number of sunshine hours (a major trigger to entrain seasonality) in the week of blood withdrawal and the 10 weeks prior to this event positively correlated with serum BDNF concentrations (Pearson’s correlation coefficients ranged: 0.05 – 0.18) and this could partly explain the observed monthly variation. These results provide strong evidence that serum BDNF concentrations systematically vary over the year. This finding is important for our understanding of those factors that regulate BDNF expression and may provide novel avenues to understand seasonal dependent changes in behavior and illness such as depression. Finally, the findings reported here should be taken into account when designing and interpreting studies on BDNF.

## Introduction

The yearly orbit of the Earth around the Sun causes variability in the length of day. Many species are sensitive to this and exhibit biochemical and behavioral alternations as response. This has been termed seasonality [1].

Seasonality has become engrained in the field of psychiatry and clinical psychology through findings such as spring peaks in suicide rates [2] and seasonal affective disorder [3], [4]. Above this, subtle effects of season on depressive behaviors have been described. For instance, in periods in which there is relatively little daylight otherwise healthy individuals show reduced levels of activity, less interest in sex, and an increased urge for sleep [5], [6]. Research at the pre-clinical level confirmed these findings by showing similar seasonal patterns in depressive-like behavior in rodents [7]–[9] and, in addition, in brain plasticity [10], [11] that has been linked to the human depressed state [12]. Taken together, these findings suggest that depressive behavior and related processes are sensitive to natural occurring environmental cues such as the length of the day.

Although the underlying mechanisms of seasonality in depressive behaviors could not be elucidated yet, some data point to variation in serotonin (5-hydroxytryptamine; 5-HT) expression as being the molecular mechanism that thrives this association [13], [14]. Indeed, human studies show that central and peripheral 5-HT activity undergoes marked seasonal rhythmicity [13]–[19]. Furthermore, 5-HT activity is positively related to the number of ambient sunshine hours [13]. However, although linked to depression, it has become increasingly clear that 5-HT alternations are not sufficient to cause the primary depressive phenotype [20]–[22]. So, seasonality in depressive behaviors might depend on changes in a pathway down-stream of 5-HT rather than directly through 5-HT signaling. Brain-Derived Neurotrophic Factor (BDNF) could serve as a component in such a pathway. BDNF is a signaling molecule that has a repertoire of regulatory functions on a related set of phenomena that are (partly) seasonal (e.g., energy balance and brain plasticity [23]–[25]). It is well established that 5-HT and BDNF interact with each other and it has been suggested that these factors together have regulatory functions in neuronal functioning and neuronal plasticity [26], [27]. Furthermore, the intertwined relationship between 5-HT and BDNF plays a fundamental role in the ‘*neurotrophin hypothesis of depression’* [28]. This hypothesis has become a leading model in the field of depression research by conceptualizing depressive disorders as being partly the consequence of deficiencies in mechanisms related to neuronal plasticity. Furthermore, the neurotrophin hypothesis predicts that BDNF and its receptor Tyrosine kinase factor B (TrkB) are targets of antidepressants because these factors modulate neuroadaptive changes that are believed to be essential for therapeutic change [12], [20], [28].

Given seasonality in 5-HT dynamics, the intrinsic relation between 5-HT and BDNF, and links between BDNF and processes and behaviors that occur according to a seasonal pattern, serum BDNF concentrations may also follow a seasonal pattern. A line of experimental work that has shown that light deprivation reduces BDNF mRNA and protein expression in the rat brain [29], [30] also gives ground to this idea. To date, however, no studies investigated seasonality in any BDNF parameter. Clarifying this issue is important in understanding the factors that influence BDNF expression, essential in evaluating research findings, and maybe helpful for a better understanding of seasonal variation in depressive-like behavior. Accordingly, we investigated seasonality in serum BDNF concentrations that are assumed to reflect central levels of BDNF [31], [32]. Since seasonality is entrained by environmental cues such as the number of sunshine hours, we also explored the relation between serum BDNF concentrations and this variable. Here we hypothesized that BDNF concentrations will be positively related to the number of sunlight hours, as has been shown for 5-HT expression [13].

## Materials and Methods

### Ethics Statement

Ethical approval for the study was obtained from the Ethical Committees of the participating Institutes and all subjects gave their written informed consent.

### Subjects

Subjects were derived from the baseline sample of the Netherlands Study of Depression and Anxiety (NESDA). The NESDA is an ongoing cohort study among 2,981 persons who were recruited in mental health care, primary care and in the general population. Included in the NESDA were persons with a depressive and/or an anxiety disorder, persons with a depressive and/or an anxiety disorder in remission and persons without lifetime depressive or anxiety disorders. Exclusion criteria were evidence for psychotic-, bipolar I- or II-, or obsessive-compulsive disorder, severe alcohol use, and not being fluent in Dutch (see Ref. [33] for full details). For our present purposes, subjects were eligible on whom data on serum BDNF concentrations and date of blood withdrawal were available (*N = *2,851, not eligible *n = *130 [∼4.5%]). Eligible and not-eligible persons did not differ with regard to gender, age and psychiatric diagnoses (all *P* values >.25).

### Demographical, Behavioral, and Clinical Measurements

Basic demographic and behavioral information on the sample (e.g., Body Mass Index (BMI measured: weight/height^2^), alcohol use, and time of blood withdrawal) was acquired through standard questions and procedures [33]. Information on the amount of physical activity the participants engaged in was acquired by means of the International physical activity questionnaire and expressed as the number of met-minutes (i.e., the ratio of the amount of energy expenditure during activity to the energy expenditure at rest) [34]. Smoking was dichotomized in current smoker versus non-current smoker and alcohol use as abstainer versus non-abstainer.

Trained staff administered the reliable and valid Composite International Diagnostic Interview version 2.1 (CIDI) [35] to establish lifetime and current (i.e., a diagnoses in the past 6-months) psychiatric diagnoses (Diagnostic and Statistical Manual of Mental Disorders, fourth edition (DSM-IV) [36]. The severity of depressive and anxiety symptoms was assessed using the Inventory of Depressive Symptoms (IDS) [37] and Beck’s Anxiety Inventory (BAI) [38]. The use of pharmacological antidepressants (i.e., selective serotonin reuptake inhibitors, serotonin-norepinephrine reuptake inhibitors, trycyclic antidepressants, noradrenergic and specific serotonergic antidepressants, and ST John’s wort) was assessed by means of self-report and drug container observation. Antidepressant use was dichotomized in yes versus no use, with use defined as intake of minimally the daily dose as recommended by the World Health Organization (WHO) [39] on >50% of the days during the last month.

### BDNF Measurements

Fifty ml of venous blood was withdrawn into vacuum tubes between 07∶30 and 09∶30 a.m. after an overnight fast (from August 2004 to March 2007). Serum was separated immediately and stored (at −85°C) until assay. Serum BDNF concentrations were determined, in a laboratory of the University of Maastricht, the Netherlands, using the Emax Immuno Assay system from Promega according to the manufacturer’s protocol (Madison, WI, USA). Absorbency was read in duplicate using a Bio-Rad Benchmark microplate reader (Hercules, CA, USA) at 450 nm. Serum BDNF concentrations were expressed as ng/ml. The coefficients of variance ranged between 2.9% and 8.1%. Further information on the exact procedures that were used and the validity and reliability of the measurements is provided elsewhere [40].

### Sunlight Measurements

The number of sunlight hours was measured using pyranometers (World Meteorological Association (WMO) [41], on a daily basis, at weather stations of the Dutch Royal Meteorological Institute (KNMI; www.knmi.nl). The weekly number of sunlight hours was defined as the sum of all sub-periods in that week for which the solar irradiance exceeded 120 W/m^2^. Since NESDA gathered data at multiple sites in the Netherlands (i.e., the Amsterdam, Groningen, and Leiden) data on the number of sunlight hours were collected from the weather stations that were most nearby the study sites (i.e., Schiphol weather station [latitude: 52°18′N] for the Amsterdam and Leiden areas [approximate latitudes: 52°31′N and 52°09′N respectively] and Eelde weather station [latitude: 53°08′N] for the Groningen area [approximate latitude: 53°12′N]).

### Statistical Analyses

Data were analyzed in SPSS 18.0 (Chicago, IL, USA) and are presented as the average ± standard deviation, unless otherwise indicated. Seasonal and monthly differences in demographical, behavioral, and clinical variables were analyzed using a Chi-square (categorical variables) or Fisher’s exact test (continuous variables). Statistical significance was set at *P*<.05 (two-tailed). Effect sizes were reported as standardized Cohen’s *d* [42], standardized regression weights (ß), or eta-squared (η^2^) where appropriate.

Analysis of covariance (ANCOVA) was used to test for differences in serum BDNF concentrations by calendar month of sampling. The following covariates were included in the analysis: gender, ethnicity, age, BMI, time of the day of blood draw, duration of serum storage, a current depression and/or anxiety diagnosis, antidepressant use, and sampling site because these variables have been shown to affect serum BDNF concentrations [40], [43]. The ANCOVA was followed up by Bonferroni corrected *post-hoc* tests when required. In view of earlier findings [40], [43], three additional sub-group analyses were performed with gender, psychiatric status, and antidepressant treatment status as additional grouping factors in order to assess potential interaction effects between these variables and month of sampling. In a similar manner we tested whether the earlier reported lower BDNF concentrations in antidepressant free depressed persons as compared to healthy control subjects, remitted depressed persons, and antidepressant treated depressed persons [43] remained statistically significant when month of sampling was controlled for.

Associations between the number of sunshine hours in the week of blood withdrawal and serum BDNF concentrations were explored using zero-order and partial Pearson’s product-moment correlation coefficients (*r*). For the latter, the variance due to the set of covariates (see above) was taken into account. We anticipated that sunlight related BDNF alternations could occur with some time delay and therefore we not only related BDNF concentrations to the number of sunshine hours in the week of blood withdrawal, but also to the number of sunshine hours in the 10 weeks prior to blood withdrawal separately. Finally, the variables coding for the number of sunshine hours in the week of blood withdrawal and the 10 weeks prior to this event, were entered together in 1 block of a multiple stepwise regression analysis (the set of covariates was entered in a first block) to test the cumulative effect of the number of sunshine hours in the recent past on serum BDNF concentrations.

## Results

The overall sample (*N = *2,851) had a mean age of 41.8±13.1 years and included 1,827 women (66.5%). Table 1 shows the characteristics of the study participants by season of sampling (Table S1 shows the characteristics of the study participants by month of sampling). Subtle seasonal differences were observed in measures for psychiatric diagnoses and depression severity. These differences were similar to the findings reported by Winthorst and colleagues [44] who studied seasonal variation of depressive and anxiety symptoms using, in part, the same data. Shortly, there was a small rise in the winter season in the percentage of clinical diagnoses of depression and depression-anxiety and related to this, on average higher scores on the IDS and a slightly higher number of persons who used an antidepressant. Next to this, persons who were sampled in the winter tended to have a somewhat higher BMI (all differences <1 BMI point) as compared to persons who were sampled in the other seasons.

**Table 1:** Descriptive information on the NESDA sample (mean ± std or percentages [*n*]) by season of sampling.

	Spring: Mar 21–Jun 20 (*n* = 693)	Summer: Jun 21–Sept 20 (*n* = 663)	Autumn: Sept 21–Dec 20 (*n* = 815)	Winter: Dec 21–Mar 20 (*n* = 680)	*P* value
Females	67.3% [466]	65.0% [431]	65.2% [531]	67.9% [462]	= .54
Age	41.5±13.2	41.0±13.3	42.5±13.1	42.3±12.5	= .08
Northern European ancestry	95.7% [663]	94.3% [625]	94.1% [766]	95.1% [647]	= .49
Education (years)	12.3±3.3	12.2±3.3	12.1±3.3	12.0±3.2	= .40
Body Mass Index	25.3±4.5	25.7±5.1	25.3±5.1	26.1±5.3	<.05 ^a^
Smoker	37.3% [258]	34.6% [229]	34.2% [279]	33.2% [226]	= .43
Physical activity ^1^	3.6±3.1	3.7±3.2	3.4±3.1	3.4±3.2	= .33
Time of blood draw	08∶48±17.4	08∶47±20.4	08∶49±33.3	08∶59±22.8	= .77
Psychiatric status					
Healthy controls ^2^	43.9% [304]	45.2% [300]	47.1% [384]	36.7% [250]	<.001^b^
MDD ^2^	12.3% [85]	12.2% [81]	11.2% [91]	16.0% [108]	<.05^a^
Anxiety ^2, 3^	20.5% [142]	18.3% [121]	17.5% [143]	21.0% [143]	= .23
Comorbid MDD-anxiety ^2, 3^	23.4% [162]	24.3% [161]	24.2% [197]	26.3% [179]	= .63
Antidepressant medication ^4^	23.4% [162]	22.2% [147]	24.9% [203]	28.2% [192]	= .05
Depression severity, IDS	21.2±14.5	20.1±14.5	20.9±15.0	22.8±13.9	<.01^a^
Anxiety severity, BAI	12.1±10.7	11.6±10.5	12.0±11.5	12.6±10.5	= .52
Non-alcohol drinker	46.4% [322]	50.9% [337]	52.2% [425]	49.5% [337]	= .21

Abbreviations: BAI, Beck’s Anxiety Inventory; IDS, Inventory of Depressive Symptoms; MDD, Major Depressive Disorder.

1 Mean met-minutes (i.e., ratio of energy expenditure during activity to energy expenditure at rest).

2 Current (6 months diagnosis).

3 Included a diagnosis of social phobia, panic disorder, generalized anxiety disorder, and/or agoraphobia.

4 Included the use of a pharmacological antidepressant (SSRI, SNRI, TCA, NaSSA, and/or St. John’s worth) for at.

least one month at regular dose (World Health Organization [WHO]).

a Post-hoc tests showed higher levels in the winter as compared to the other seasons.

b Post-hoc tests showed higher percentages in the summer, autumn and spring as compared to the winter season.

### Seasonality in Serum BDNF Concentrations

**Figure 1:**
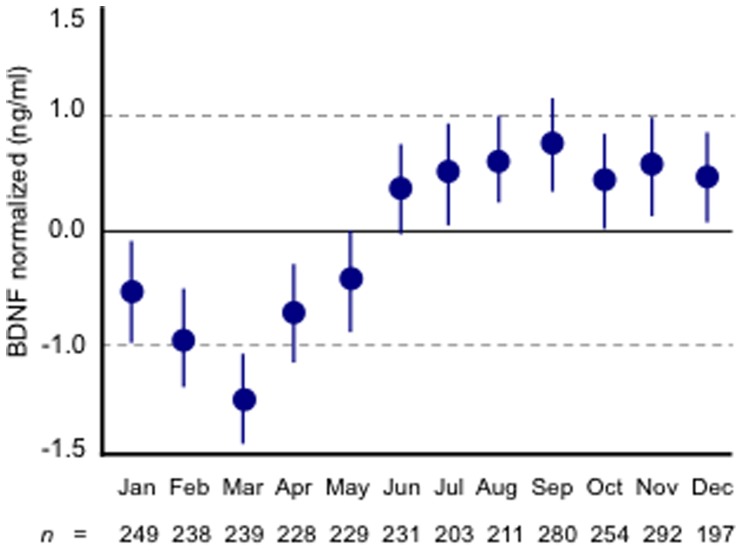
Serum BDNF concentrations by month of sampling. Error bars reflect the SEM. For pair-wise comparisons we refer to Table S2.

**Figure 2:**
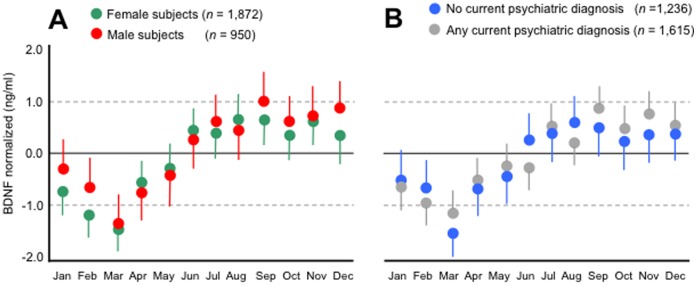
Serum BDNF concentrations by month of sampling and gender (Panel A) and current psychiatric status (Panel B). Error bars reflect the SEM.

Using ANCOVA we found strong indications for a seasonal pattern in serum BDNF concentrations (overall *F = *11.32, *P*<.0001, η^2^ = 0.04, see Figure 1). This seasonal pattern was such that serum BDNF concentrations were, on average, lower in the months January to May and higher in the months June to December. Pair-wise comparisons by month of sampling are given in Table S2. Effect sizes for monthly differences that were statistically significant after Bonferroni corrections ranged from small (*d = *0.27, January versus August) to large (*d = *0.66, March versus September). Results from separate ANCOVA’s showed a similar seasonal pattern in serum BDNF concentrations for males and females (see Figure 2, panel A), depressed and non-depressed persons (see Figure 2, panel B), and for antidepressant treated (any antidepressant) and antidepressant free persons (data not shown). *P* values for the interaction terms between these additional grouping factors and month of sampling were all >.10. Given our earlier findings showing that serum BDNF concentrations were highest in currently depressed persons on selective serotonin reuptake inhibitors or ST John’s wort [43] we in addition tested for seasonality in serum BDNF concentrations in persons using these agents. Here, a similar seasonal effect in serum BDNF concentrations was observed (η^2^ = 0.074).

Nadir (March) and peak (September) BDNF concentrations showed a correspondence with the end of the Dutch winter and the end of the Dutch summer respectively. Even more, the pattern of BDNF concentrations over the year suggested an increase during the Dutch spring-summer period (equinox vernal, March 20 to September 22) and a decrease in the Dutch autumn-winter period (equinox autumnal, September 23 to March 19). Indeed, when decomposed by equinox, we observed a coherent pattern of increasing BDNF concentrations during the spring and summer (*n = *1,356, ß = 0.19, *P*<.0001) and decreasing BDNF concentrations during the autumn and winter (*n = *1,495, ß = −0.17, *P*<.0001; see Figure S1; *t = *9.87 (*P*<.0001) for the interaction equinox of sampling - consecutive week of sampling within the equinox).

Although somewhat attenuated, the earlier reported main effects [43] of lower serum BDNF concentrations in antidepressant free depressed persons as compared to healthy control subjects, remitted depressed persons, and antidepressant treated depressed persons [43] remained statistically significant (*P = *.04, *P*<.001, and *P = *.04 respectively) in analyses in which these factors coding for diagnostic and treatment status were added as grouping factors next to month of sampling.

### Explorative Analyses: Sunshine Hours and Serum BDNF Concentrations

The number of sunshine hours per week throughout the year ranged from 2 hours per week to 131 hours per week. Exploratory analyses showed small positive correlations between the number of sunshine hours (in the week of blood withdrawal, and the 10 weeks prior to that) and serum BDNF concentrations ([zero order and partial] in the range: *r* = 0.04 – *r* = 0.18; see Table S3 and Figure S2). Correlations were largest for the number of sunlight hours 7 and 8 weeks prior to blood withdrawal. Together, the number of weekly sunlight hours in the week of sampling and in the 10 weeks prior to the week of sampling, positively predicted serum BDNF concentrations (entered in 1 block of a multiple stepwise regression analysis (the set of covariates was entered as a first block): Δ*F = *11.22, multiple correlation = 0.21, *P*<.0001). The number of sunshine hours could partly explain the monthly differences in serum BDNF concentrations (i.e., η^2^ = 0.041, *P*<.0001 before and η^2^ = 0.011, *P = *.003 after the inclusion of the number of sunlight hours as covariates in the ANCOVA). Additional explorative analyses showed a similar pattern of correlations between the length of the natural photoperiod in the week of blood withdrawal, and the 10 weeks prior to that, and serum BDNF concentrations.

## Discussion

The novel finding that emerges from this study is that of pronounced seasonal variation in serum BDNF concentrations. The seasonality that we observed followed a coherent pattern of increasing BDNF concentrations over the course of the spring and the summer and, conversely, decreasing BDNF concentrations over the course of the autumn and the winter. Illustrative for the robustness of our finding was that the seasonal pattern was similar for both sexes and for conditions in which BNDF expression is altered, such as a diagnosis of major depression [45]. By means of covariate adjusted analyses we could exclude a range of potentially alternative explanations for our findings, including effects of for example physical activity and BMI that have been reported to be seasonal [46], [47] and that have been linked to serum BDNF concentrations as well [48], [49]. In addition, our results were of a relatively large magnitude with effect sizes (Cohen’s *d*) up to 0.66 for monthly differences. Together, these findings may have significant implications as discussed below.

### Seasonality in Serum BDNF Concentrations: Mechanisms and Theoretical Implications

What might explain the strong pattern of seasonality in serum BDNF concentrations? Differences in cAMP-response element binding protein (CREB) activity could be an option. CREB is a transcription factor that binds to the promoter region of the BDNF gene and positively regulates BDNF transcription [50]. CREB activity is under the influence of 5-HT. Given that long day conditions give rise to a higher 5-HT expression [13], seasonality in serum BDNF concentrations may be entrained by 5-HT induced CREB activation. This explanation would fit with observations that come from the field of antidepressant research. For example, increases in the transcriptional activity of BDNF in the course of treatment with a selective serotonin reuptake inhibitor are often observed but these only occur in the face of an increase in CREB activity [51]. Also, antidepressants give rise to increased CREB activity but only if they are administered chronically (e.g., ≥21 days) [51]. This appears to be in agreement with our observation that the correlations between BDNF concentrations and the number of sunshine hours were largest for the number of sunshine hours in 7 to 8 weeks prior to blood withdrawal. Furthermore, peak serum BDNF values were observed in the early autumn and nadir values in the early spring. So, the seasonality in BDNF expression seems to occur with a time-delay relative to the seasons and their corresponding weather characteristics.

Important for the interpretation of our results is the question whether peripheral differences in serum BDNF concentrations imply that there also are differences at the level of the Central Nervous System (CNS). As already shortly mentioned in the introduction part of our paper, it has been shown in rodents that BDNF crosses the blood-brain barrier [31] and that peripheral and central BDNF concentrations correlate positively in rodents and in pigs [32]. Therefore it is reasonable to assume that blood BDNF concentrations reflect BDNF concentrations in the CNS. Unfortunately the literature on this topic in humans is limited to only one study in which blood simultaneously was derived from high up the jugular veins and arterial veins [52]. The results of this study showed that BDNF levels were higher in blood that was derived from the internal jugular veins as compared to arterial blood. This indeed is indicative for CNS production of BDNF that is obtained from peripheral tissues [52]. It should be noted though that some studies report null-findings with regard to an association between peripheral BDNF concentrations and more central parameters for BDNF activity (e.g., the absence correlations between plasma and cerebral spinal fluid concentrations of BDNF [53]).

### Seasonality in Serum BDNF Concentrations: Practical Implications

Notwithstanding some uncertainty with regard to the exact mechanisms underlying the seasonality in BDNF expression, our findings have immediate and important practical implications. First, our findings are of essential importance in the interpretation of results from longitudinal studies. That is, the results of trials that have serum BDNF concentrations as an outcome measure and that span several months, might be of little use unless detailed knowledge on seasonal effects is available. Second, and in agreement with the latter statement, our results stress the need to sample groups (e.g., depressed patients versus healthy control subjects) equally over the year in order to gain credibility and validity in research findings.

### Strengths, Limitations, and Future Studies

Our study has strengths, notably reliability through a large sample size and validity through the adjustment for a range of confounders and the possibility to perform subgroup analyses. However, there are also limitations. First, we used between-subject data whereas within-subject data with repeated samplings (monthly or more often) over at least 1 calendar year is more appropriate to establish seasonality. Second, there must have been some noise in the measurements related to the variables that coded for the hours of sun in the weeks prior to blood withdrawal. That is, we assigned each individual the number of sun hours that were recorded in the particular region these persons lived in under the assumption that they actually were in that particular region. However, we do not know this with certainty (e.g., participants may have been on vacation) and we also do not know whether they truly were exposed to the sun (e.g., participants may have stayed inside). We assume that this resulted in random noise that decreased our ability to detect associations and thus that the true associations between sunlight hours and serum BDNF concentrations are likely to be of a larger magnitude than as reported here. Also, from our data we can not conclude whether serum BDNF concentrations are kept in a certain homeostatic range over the year to serve a given function or whether we observed an epiphenomenon of some other physiological or (unmeasured) behavioral process that may be unrelated to central BDNF functioning. Finally, a limitation with regard to the Promega BDNF ELISA kit that was used in our study for serum BDNF measurements is that it quantifies total amount of BDNF without distinguishing between the pro- and the mature BDNF forms [54], [55]. It could well be that the percentage of mature BDNF to total BDNF in serum may be altered as a function of seasonal period. Only very recently it has been shown that such an important distinction can be made [55], [56] and thus future studies could look into differences in pro-mature BDNF ratios over the seasons.

For future studies it further would be interesting to elucidate whether tryptophan, 5-HT, and/or CREB activity, truly have important roles, as hypothesized here, in the chain of events that in the end may lead to seasonality in serum BDNF concentrations. It also would be particularly interesting to study BDNF concentrations as a function of selected levels of (sun)light exposure. We would expect that serum BDNF concentrations will vary less in areas where the seasons, the number of sunlight hours, and/or the natural length of day vary less over the year (e.g., closer to the equator as compared to the Netherlands or 12 hours light/dark regimens in laboratories). Finally, in the light of our findings that serum BDNF concentrations are correlated with the amount of ambient sunlight (and with the length of the photoperiod) an important area of investigation would be to investigate changes in serum BDNF concentrations in the course of treatment with bright light [57] in conditions that have been associated with an altered BDNF expression [30].

### Summary and Conclusions

Here we demonstrate that serum BDNF concentrations profoundly vary over the year and that this occurs according to a coherent pattern of increasing BDNF concentrations in the spring-summer period and decreasing BDNF concentrations in the autumn-winter period. In addition we show correlations between serum BDNF concentrations and the number of sunlight hours. Although much remains to be understood with regard to these associations and notwithstanding some limitations, we believe that these results invite for a perspective on BDNF related mechanisms in which seasonality is engrained.

## Supporting Information

Figure S1 **Mean normalized serum BDNF concentrations plotted as a function of each consecutive week of measurement in each equinox.**
(TIF)

Figure S2 **Partial Pearson’s correlation coefficients (for covariates see the paper) on the relation between mean serum BDNF concentrations and the hours of sunlight in the week of blood draw (wk-0) and the 10 weeks prior to blood draw (wk-1 to wk-10).** *P<.05, **P<.001, ***P<.0001
(TIF)

Table S1 **Descriptive information on the sample (mean ± std or percentages [** ***n*** **]) by month of sampling.**
(DOC)

Table S2 ***P*** **values for pair-wise comparisons on covariate adjusted serum BDNF concentrations by month of sampling.**
(DOC)

Table S3 **Zero-order and partial Pearson’s product-moment correlation coefficients and corresponding** ***P*** **values on the associations between the number weekly sunlight hours and serum BDNF concentrations.**
(DOC)
