# Supplementary figures and images for: Serum BDNF Concentrations Show Strong Seasonal Variation and Correlations with the Amount of Ambient Sunlight
Source: PLoS One. 2012 Nov 2;7(11):e48046. doi: 10.1371/journal.pone.0048046 (PMC3487856; doi:10.1371/journal.pone.0048046)

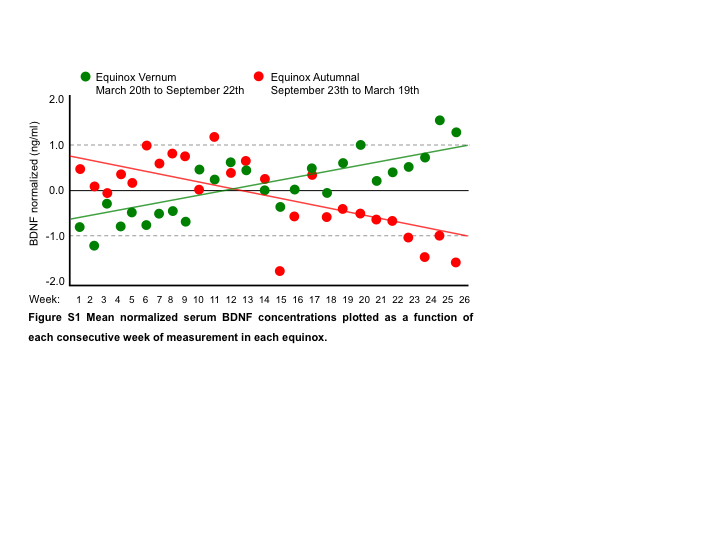

Supplement: Figure S1 — Mean normalized serum BDNF concentrations plotted as a function of each consecutive week of measurement in each equinox. (TIF) [file pone.0048046.s001.tif]

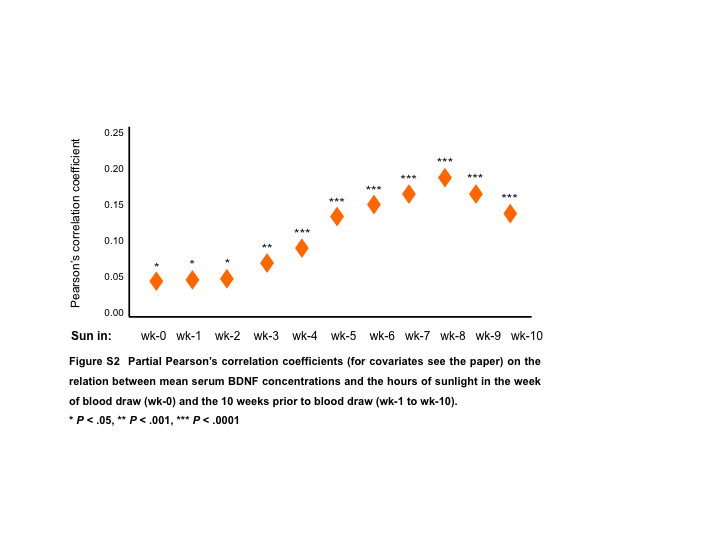

Supplement: Figure S2 — Partial Pearson’s correlation coefficients (for covariates see the paper) on the relation between mean serum BDNF concentrations and the hours of sunlight in the week of blood draw (wk-0) and the 10 weeks prior to blood draw (wk-1 to wk-10). *P<.05, **P<.001, ***P<.0001 (TIF) [file pone.0048046.s002.tif]
