# Supplementary material for: Serum BDNF Concentrations Show Strong Seasonal Variation and Correlations with the Amount of Ambient Sunlight
Source: PLoS One. 2012 Nov 2;7(11):e48046. doi: 10.1371/journal.pone.0048046 (PMC3487856; doi:10.1371/journal.pone.0048046)
Supplement: Table S1 — Descriptive information on the sample (mean ± std or percentages [ n ]) by month of sampling. (DOC) [file pone.0048046.s003.doc]

| **Table S2 *P* values for pair-wise comparisons on covariate adjusted serum BDNF concentrations by month of sampling** | | | | | | | | | | | | |
| --- | --- | --- | --- | --- | --- | --- | --- | --- | --- | --- | --- | --- |
|  | Jan  *n* = 249 | Feb  *n* = 238 | Mar  *n* = 239 | Apr  *n* = 228 | May  *n* = 229 | Jun  *n* = 231 | Jul  *n* = 203 | Aug  *n* = 211 | Sep  *n* = 280 | Oct  *n* = 254 | Nov  *n* = 292 | Dec  *n* = 197 |
| Jan | 1 | .19 | .01 ↑ | .74 | .26 | .001 ↓ | .003 ↓ | .001*↓ | <.001*↓ | .004 ↓ | <.001*↓ | .003 ↓ |
| Feb | .19 | 1 | .21 | .35 | .02 ↓ | <.001*↓ | <.001*↓ | <.001*↓ | <.001*↓ | <.001*↓ | <.001*↓ | <.001*↓ |
| Mar | .01 ↓ | .21 | 1 | .03 ↓ | <.001*↓ | <.001*↓ | <.001*↓ | <.001*↓ | <.001*↓ | <.001*↓ | <.001*↓ | <.001*↓ |
| Apr | .74 | .35 | .03 ↑ | 1 | .15 | <.001*↓ | .001 ↓ | <.001*↓ | <.001*↓ | .001 ↓ | <.001*↓ | .002 ↓ |
| May | .26 | .02 ↑ | <.001*↑ | .15 | 1 | .04 ↓ | .06 | .02 ↓ | .001 ↓ | .09 | .006 ↓ | .07 |
| Jun | .001 ↑ | <.001*↑ | <.001*↑ | <.001*↑ | .04 ↑ | 1 | .92 | .78 | .27 | .69 | .59 | .87 |
| Jul | .003 ↑ | <.001*↑ | <.001*↑ | .001 ↑ | .06 | .92 | 1 | .72 | .25 | .78 | .53 | .94 |
| Aug | .001*↑ | <.001*↑ | <.001*↑ | <.001*↑ | .02 ↑ | .78 | .71 | 1 | .44 | .50 | .81 | .66 |
| Sep | <.001*↑ | <.001*↑ | <.001*↑ | <.001*↑ | .001 ↑ | .27 | .25 | .44 | 1 | .12 | .56 | .22 |
| Oct | .004 ↑ | <.001*↑ | <.001*↑ | .001 ↑ | .09 | .69 | .78 | .50 | .12 | 1 | .33 | .84 |
| Nov | <.001*↑ | <.001*↑ | <.001*↑ | <.001*↑ | .006 ↑ | .59 | .53 | .82 | .56 | .33 | 1 | .48 |
| Dec | .003 ↑ | <.001*↑ | <.001*↑ | .002 ↑ | .07 | .87 | .94 | .66 | .22 | .84 | .48 | 1 |
| * Statistically significant after Bonferroni correction was applied (66 comparisons, critical *P* value = .00076 )  ↑ Higher serum BDNF levels in the month indicated in the row relative to the month indicated in the corresponding column  ↓ Lower serum BDNF levels in the month as indicated in the row relative to the month as indicated in the corresponding column | | | | | | | | | | | | |
