# Supplementary material for: Serum BDNF Concentrations Show Strong Seasonal Variation and Correlations with the Amount of Ambient Sunlight
Source: PLoS One. 2012 Nov 2;7(11):e48046. doi: 10.1371/journal.pone.0048046 (PMC3487856; doi:10.1371/journal.pone.0048046)
Supplement: Table S2 — P values for pair-wise comparisons on covariate adjusted serum BDNF concentrations by month of sampling. (DOC) [file pone.0048046.s004.doc]

| **Table S3 Zero-order and partial Pearson’s product-moment correlation coefficients and corresponding *P* values on the associations between the number weekly sunlight hours and serum BDNF concentrations** | | | | |
| --- | --- | --- | --- | --- |
|  | Zero-order *r* | *P* value | Partial *r* ^1^ | *P* value |
| Number of sunlight hours in the: |  |  |  |  |
| Week of blood draw | 0.03 | .08 | 0.04 | .03 |
| Week prior to blood draw | 0.03 | .07 | 0.04 | .04 |
| Two weeks prior to blood draw | 0.02 | .11 | 0.04 | .03 |
| Three weeks prior to blood draw | 0.04 | .01 | 0.06 | .001 |
| Four weeks prior to blood draw | 0.07 | <.0001 | 0.09 | <.0001 |
| Five weeks prior to blood draw | 0.12 | <.0001 | 0.13 | <.0001 |
| Six weeks prior to blood draw | 0.14 | <.0001 | 0.15 | <.0001 |
| Seven weeks prior to blood draw | 0.15 | <.0001 | 0.16 | <.0001 |
| Eight weeks prior to blood draw | 0.16 | <.0001 | 0.18 | <.0001 |
| Nine weeks prior to blood draw | 0.15 | <.0001 | 0.16 | <.0001 |
| Ten weeks prior to blood draw | 0.12 | <.0001 | 0.13 | <.0001 |
| ^1^ See the paper for covariates | | | | |
